# COVID-19 and Cardiomyopathy: A Systematic Review

**DOI:** 10.3389/fcvm.2021.695206

**Published:** 2021-06-17

**Authors:** Fatemeh Omidi, Bahareh Hajikhani, Seyyedeh Neda Kazemi, Ardeshir Tajbakhsh, Sajedeh Riazi, Mehdi Mirsaeidi, Ali Ansari, Masoud Ghanbari Boroujeni, Farima Khalili, Sara Hadadi, Mohammad Javad Nasiri

**Affiliations:** ^1^Department of Cardiology, Imam Hossein Hospital, Shahid Beheshti University of Medical Sciences, Tehran, Iran; ^2^Department of Microbiology, School of Medicine, Shahid Beheshti University of Medical Sciences, Tehran, Iran; ^3^Preventative Gynecology Research Center, Imam Hossein Hospital, Shahid Beheshti University of Medical Sciences, Tehran, Iran; ^4^Anesthesiology Research Center, Imam Hossein Hospital, Shahid Beheshti University of Medical Sciences, Tehran, Iran; ^5^Imam Hossein Hospital, Shahid Beheshti University of Medical Sciences, Tehran, Iran; ^6^Department of Pulmonary and Critical Care, University of Miami Miller School of Medicine, Miami, FL, United States; ^7^School of Medicine, Shahid Beheshti University of Medical Sciences, Tehran, Iran

**Keywords:** COVID-19, cardiomyopathy, cardiac injury and regeneration, systematic review, SARS-CoV-2

## Abstract

**Background:** Cardiomyopathies (CMPs) due to myocytes involvement are among the leading causes of sudden adolescent death and heart failure. During the COVID-19 pandemic, there are limited data available on cardiac complications in patients with COVID-19, leading to severe outcomes.

**Methods:** We conducted a systematic search in Pubmed/Medline, Web of Science, and Embase databases up to August 2020, for all relevant studies about COVID-19 and CMPs.

**Results:** A total of 29 articles with a total number of 1460 patients were included. Hypertension, diabetes, obesity, hyperlipidemia, and ischemic heart disease were the most reported comorbidities among patients with COVID-19 and cardiomyopathy. In the laboratory findings, 21.47% of patients had increased levels of troponin. Raised D-dimer levels were also reported in all of the patients. Echocardiographic results revealed mild, moderate, and severe Left Ventricular (LV) dysfunction present in 17.13, 11.87, and 10% of patients, respectively.

**Conclusions:** Cardiac injury and CMPs were common conditions in patients with COVID-19. Therefore, it is suggested that cardiac damage be considered in managing patients with COVID-19.

## Introduction

The emergence of severe acute respiratory syndrome coronavirus 2 (SARS-CoV-2), which was first reported on 31 December 2019 from Wuhan, China, resulted in an unprecedented outbreak of Coronavirus disease 2019 (COVID-19). The most common manifestation of COVID-19 is pulmonary complications. However, this novel disease's presentations have a broad spectrum of signs and symptoms from asymptomatic infection or mild flu-like symptoms to multiorgan failure resulting in death (1, 2). Cardiovascular disease (CVD) has been reported in patients infected with COVID-19 (3). Based on the literature, 20–30% of hospitalized patients showed cardiovascular manifestations associated with worse outcomes (4, 5). Cardiovascular complications of COVID-19

are thought to be a combination of direct viral injury and the host's immune response resulting in vascular inflammation, plaque instability, and myocardial inflammation (6–9). Cardiomyopathies (CMPs) which resulted from heart muscle involvement, are among the main causes of adolescent sudden death and heart failure (10). SARS-CoV-2 infection in patients suffering from CMPs represents an actual risk of exacerbating patient clinical status (11).

Although many authors have reported various aspects of respiratory-related symptoms of COVID-19, the increasing prevalence of cardiac complications in COVID-19 patients should be taken into considerations (12–17). Thus, this study was aimed to systematically review the current published literature to evaluate clinical and paraclinical characteristics of CMPs in patients infected with SARS-CoV-2.

## Methods

### Search Strategy

In the following bibliographic databases, we carried out a comprehensive systematic search of literature: PubMed/Medline, Embase, and Web of Science. We searched for any relevant articles published in English up to August 2020. The search included keywords including COVID-19, severe acute respiratory syndrome coronavirus 2, SARS-CoV-2, in combinations with cardiomyopathy, or CMP, cardiomyopathies, myocardiopathy, cardiac injury, or myocarditis.

Additionally, all references of selected papers were searched manually for additional related articles. The present systematic review conforms to the “Preferred Reporting Items for Systematic Reviews and Meta-Analyses” (PRISMA) statement (18).

### Study Selection

Studies reported any data about CMPs in patients with confirmed COVID-19 were included. Abstracts, commentary, letter to editor, guidelines, and review articles were excluded.

All retrieved publications were screened for eligibility in two phases. First, two reviewers independently screened the titles and abstracts of potentially relevant articles identified in the primary search. Subsequently, a review of the full texts of all remaining articles was done by the same authors. Any discrepancy in the article selection or technical uncertainties were discussed and resolved between review authors.

#### Data Extraction

The following variables were extracted from all included studies: first author, year of publication, type of study, country where the research was conducted, study population, COVID-19 diagnosis technique, laboratory findings, treatment protocols, and type of CMPs. Two authors independently extracted the data from the selected studies. The data was jointly reconciled, and disagreements were discussed and resolved between review authors.

## Results

**Figure 1:**
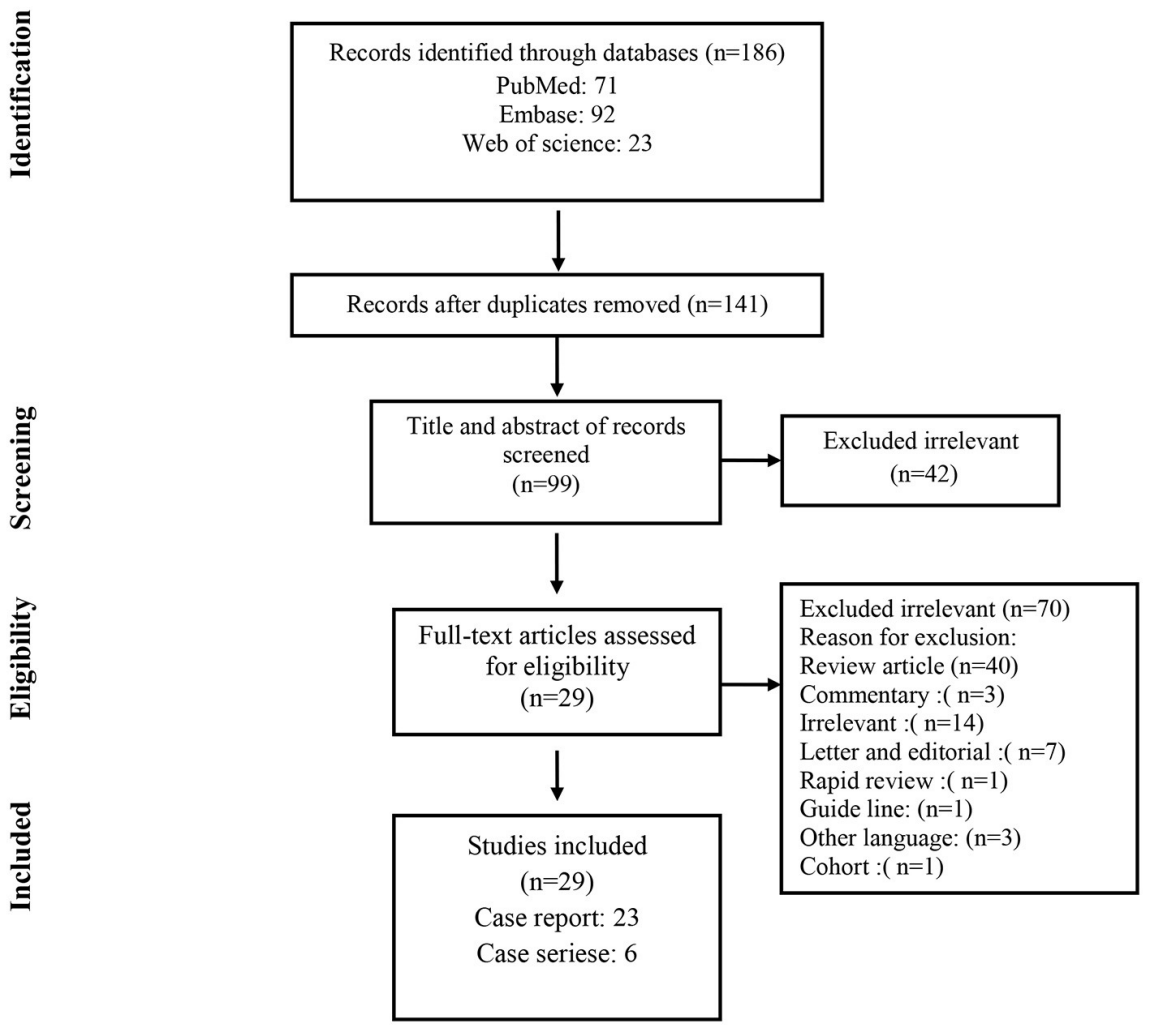
Flow chart of study selection for inclusion in the systematic review.

As shown in Figure 1, a total of 186 studies were identified from databases. After removing 45 duplicates, 141 non-duplicate studies remained for further assessments. After applying the inclusion/exclusion criteria, 29 articles (22 case reports and 7 case series) were included with a total number of 1460 unique cases of COVID-19 with a mean age of 58 years. The characteristics of the included studies are described in Table 1.

**Table 1:** Characteristics of the included studies.

**References**	**Country**	**Type of study**	**No. of patients**	**Male/female**	**Mean age**
Doyen et al. (19)	Italy	Case report	1	1 M	69
Paul et al. (20)	France	Case report	1	1 M	35
Huyut (21)	Turkey	Case report	1	1 F	59
Pasqualetto et al. (22)	Italy	Case series	3	2 M-1 F	83.33
Deng et al. (23)	China	Case series	14	10 M-4 F	74
Taza et al. (24)	USA	Case report	1	1 M	52
Roca et al. (25)	Italy	Case report	1	1 F	87
Minhas et al. (26)	USA	Case report	1	1 F	52
Juusela et al. (27)	USA	Case series	2	2 F	35.5
Meyer et al. (28)	Switzerland	Case report	1	1 F	83
Khalid et al. (29)	Italy	Case report	1	1 F	76
Nguyen et al. (30)	Belgium	Case report	1	1 F	71
Bonnet et al. (31)	France	Case report	1	1 M	27
Zhang et al. (32)	Multicenter	Case series	2	1 M-1 F	59
Dabbagh et al. (33)	USA	Case report	1	1 F	67
Guo et al. (34)	China	Case series	187	91 M-96 F	58.5
Tavazzi et al. (35)	Italy	Case report	1	1 M	69
Hua et al. (36)	UK	Case report	1	1 M	47
Villanueva et al. (37)	USA	Case report	1	1 M	68
Kir et al. (38)	USA	Case report	1	1 M	49
Dweck et al. (39)	Multicenter	Case series	1209	844 M-365 F	62
Irabien-Ortiz et al. (40)	Spain	Case report	1	1 M	59
Craver et al. (41)	USA	Case report	1	1 M	17
Bobeck et al. (42)	USA	case report	1	1 M	80
Arentz et al. (43)	USA	Case series	21	10 M-11 F	70
Yildirim and Karaagac (44)	Turkey	Case report	1	1 F	7
Chadha (45)	USA	Case report	1	1 F	85
Kim et al. (46)	Korea	Case report	1	1 F	21
Luetkens et al. (47)	Germany	Case report	1	1 M	79

Table 2 shows the outcomes and prognosis of CMPs in patients with COVID-19. 98 out of 1,212 evaluated patients developed cardiogenic shock (8.08%). Six studies reported mortality rates, showing 48 out of 192 (25%) of patients deceased.

**Table 2:** The outcomes and prognosis of CMPs.

**Outcomes**	**No of study**	***n/N***	**Percentage (%)**
Deceased	6	48/192	25
Cured	13	14/16	87.5
**Prognosis**
Cardiogenic shock	4	98/1,212	8.08
MOD^a^	3	81/191	42.40
ARDS^b^	8	67/215	31.16

*n, number of patients with any variables; N, the total number of studied patients*.

a *MOD,Multi organ disease;*

b *ARDS, Adult respiratory distress syndrome*.

As presented in Table 3, hypertension, diabetes, obesity, hyperlipidemia, ischemic disease, and obstructive sleep apnea were the most reported comorbidities among them.

**Table 3:** Clinical and laboratories findings in patients with COVID-19.

	**Variable**	**No of study**	***n/N***	**%**
Clinical manifestations	Chest pain	9	128/1,232	10.38
	Dyspnea	9	9/10	90
	Shortness of breath	11	39/45	88.66
	Cough	14	38/49	77.55
	Fever	14	40/51	78.43
	Fatigue	5	5/5	5
	Tachypnea	7	8/21	30.09
	Crackles	7	7/8	87.5
	Diarrhea	3	3/3	100
	Nausea & vomiting	4	5/6	83.33
Signs	Elevated pulse rate	12	195/1,413	13.8
	Elevated temperature	12	32/33	96.96
Comorbidities	Hypertension	15	526/1,424	36.93
	Diabetes	10	279/1,440	19.37
	Obesity	3	11/17	64.7
	Hyperlipidemia	5	5/5	100
	Ischemic disease	4	200–1,431	13.97
	Obstructive sleep apnea	2	10/35	25.57
	COPD^a^	3	12/222	5.4
	CKD^b^	3	17/209	8.13
	CA^c^	3	15/189	7.93
Laboratory findings	elevated NTproBNP	11	26/214	12.2
	High IL-6	5	5/5	100
	High D-dimer	5	6/6	100
	High ferritin	6	6/6	100
	High CRP^d^	14	14/201	6.96
	High Troponin	18	307/1412	21.74

*n, number of patients with any variables; N, the total number of studied patients.*

a *COPD, Chronic obstructive pulmonary disease;*

b *CKD, Chronic kidney disease;*

c *CA, Copd/Asthma;*

d *CRP, C-reactive protein*.

As shown in Table 3, cough and fever were reported as the most prevalent symptoms in 14 out of 29 studies. Dyspnea was reported in 9 studies. According to these studies, 90% of the evaluated patients had this complication. Evaluation of laboratory findings showed elevated troponin levels in 18 studies with 308 out of 1,412 patients (21.47%). Increased D-dimer levels were reported in 5 case reports, of which six patients showed this elevated marker.

CMPs evidence in patients with COVID-19 indicates in Table 4. Common ECG findings were: tachycardia, premature beats, ST-segment elevation, blocks, and inverted T wave. Inverted T waves were seen in EKG findings of 9 studies (91.66% of evaluated patients). Left ventricular (LV) involvement is a hallmark of primary CMPs. Echocardiographic findings revealed mild (17.1%), moderate (11.85%), and severe (9.98%) LV dysfunction, which was discussed in 6, 4, and 11 studies, respectively. Aneurysm formation, a sign of stress-induced cardiomyopathy followed COVID-19, was found in all 11 evaluated patients (100%). Regional wall motion abnormalities (RWMA) as another sign were found in 46/1,217 (1.15%) patients. Right ventricular (RV) involvement and high pulmonary artery pressure (PAP) are signs of the destruction of the right heart. RV enlargement was presented in 14.88% of tested patients (181/1,216). RV dysfunction was also found in 26.01% (315/1,211) of patients' echocardiograms. Findings of Chest X-Ray (CXR) and Chest CT scan showed ground-glass opacification (GGO) patterns (26 of 34 patients) and consolidation (7 of 7 patients) as the most common findings (Table 4). Among the type of CMPs, COVID CMPs, and hypertrophic cardiomyopathy were among the most reported type in 39.13 and 18.75% cases, respectively.

**Table 4:** Cardiomyopathy evidence in patients with COVID-19.

	**Variable**	**No of study**	***n/N***	**%**
EKG	Sinus tachycardia	7	8/8	100
	Bradycardia	2	2/2	100
	Premature beats	2	4/4	100
	ST elevation	5	5/5	100
	ST depression	2	2/2	100
	Blocks	2	4/4	100
	Inverted T wave	9	11/12	91.66
	VT^a^	2	49/1,403	3.49
Echocardiography	LVE (LV^b^ enlargement)	3	68/1,219	5.57
	Mild LV dysfunction	6	208/1,216	17.10
	Moderate LV dysfunction	4	144/1,215	11.85
	Severe LV dysfunction	11	122/1,222	9.98
	RVE (RV^c^ enlargement)	1	181/1,216	14.88
	RV dysfunction	3	315/1,211	26.01
	High PAP^d^	1	99/1,216	8.14
	Aneurysm formation	10	11/11	100
	RWMA^e^	10	46/1,217	1.15
	Pericardial effusion	3	3/3	100
	LVH^f^	4	4/4	100
	Pericardial effusion	3	3/3	100
	Endocarditis	1	14/1,216	1.15
	Tamponade	3	13/1,218	1.06
	Echo MI^g^	2	37/1,230	3
	Echo Myocarditis	1	35/1,216	2.87
	D shap LV	1	49/1,216	4.02
CXR	Diffuse involvement	6	7/7	100
	Cardiomegaly	2	2/3	66.66
CT scan	Ground-glass opacities	11	26/34	76.47
	Consolidation	5	7/7	100
Angiogram	Abnormal angiogram	5	6/7	85.71
	Normal angiogram	1	1/1	100
Type of cardiomyopathy	DCM^h^	3	71/1,225	5.79
	HCM^i^	3	3/16	18.75
	Myocarditis	8	55/1,229	4.47
	Myocardial injury	13	303/1,408	2.3
	Takotsubo	14	32/1,222	2.61
	Ischemic after COVID	1	36/1,216	2.96
	COVID cardiomyopathy	3	9/23	39.13

*n, number of patients with any variables; N, the total number of studied patients.*

a *VT, Ventricular tachycardia;*

b *LV, Left ventricular;*

c *RV, Right ventricular;*

d *PAP, Pulmonary artery pressure;*

e *RWMA, Regional wall motion abnormalities;*

f *LVH, Left ventricular hypertrophy;*

g *M, myocardial infarction;*

h *DCM, Dilated cardiomyopathy;*

i *HCM, Hyper trophic cardiomyopathy*.

In terms of treatment, 10 out of 14 patients (71.42%) reported in 11 studies received β-Blocker as part of their treatment regimen. The use of Diuretic agents was reported in 7 studies which included 7 out of 9 (77.77%) patients (Table 5).

**Table 5:** Treatment agents used in the included studies.

		**Variable**	**No of study**	***n/N***	**%**
Non-pharmacologic treatment		O2 nasal	8	10/11	90.9
		Intubation	14	72/223	32.28
		Pericardiocentesis	3	3/3	100
Pharmacologic treatment	Antimicrobial agents	Antibacterial drugs	6	188/193	97.4
		Azithromycin	6	6/7	85.71
		Antiviral drugs	4	171/192	89.06
	Immunomodulators	Hydroxychloroquine	9	10/12	83.33
		IVIG^a^	3	23/189	12.16
		steroid	8	113/194	58.24
		Tocilizumab	4	4/5	80
	Anticoagulant	Fondaparinux	3	4/5	80
		Anti-platelet	3	4/5	80
		Heparin/LMWH^b^	6	6/7	85.71
	Others	ACE/ARB^c^	4	4/4	100
		β-Blocker	10	10/14	71.42
		NEP^d^	5	5/6	83.33
		Diuretic	7	7/9	77.77
		Vasopressor	5	5/5	100

a *IVIG, Intravenous immune globulin;*

b *LMWH, Low molecular weight heparin;*

c *ACE/ARB, angiotensin converting enzyme inhibitors/angiotensin-receptor blockers;*

d *NEP, Norepinephrine*.

## Discussion

COVID-19 has resulted in other organ involvement, and CMPs are among the most significant complications of this rapidly emerging disease, causing more severe disease and increased mortality rates (48, 49). In this systematic review, we studied the cardiac injuries in patients with SARS-CoV-2 infection that resulted in CMPs. Echocardiographic results showed a range of mild to severe left ventricular dysfunction in 10% to 17.13% of the studied patients.

The patients' recovery and death rates were assessed in 20 studies that showed that 28.7% of patients with one type of CMPs died following SARS-CoV-2 infection. Patients with cardiovascular comorbidities had a higher risk of developing cardiac injury (50).

In a study on twenty-one critically ill patients admitted in intensive care units (ICU), one-third developed CMPs (51). Yang et al. showed 52 critically ill COVID-19 patients 12 (23%) presented with cardiac injury (52).

The results of a cohort study showed that 23% of patients experienced new heart failure or exacerbation of chronic heart failure, of which 28 survived, and 16 died (50).

Based on the included studies that examined patients' mortality rate with CMPs and COVID-19, 25% of these patients were deceased. As a result, it can be inferred that cardiac injury is a significant predisposing factor for increasing the mortality rate of COVID-19.

Huang et al. demonstrated a “Cytokine storm” model that results in a pro-inflammatory markers surge that may lead to myocardial injury (53). Similar effects have been observed with MERS-CoV and SARS-CoV infections previously (54). Furthermore, the virus may be involved in a primary myocardial injury by entering the myocytes through the ACE-2 receptor (55).

Overall, SARS-CoV-2 can cause cardiac complications through the following pathways: (1) Indirect cardiac injury due to increased release of cytokines and inflammatory pathways. (2) Direct invasion of the SARS-COV-2 in cardiac myocytes. (3) Respiratory damage can cause hypoxia, myocardial supply-demand mismatch, followed by oxidative stress and damage to cardiomyocytes (56, 57).

There are different manifestations of cardiac involvement in COVID-19, including acute myocardial infarction, acute heart failure, cardiogenic shock, myocarditis, and fatal arrhythmias (58). Myocardial injury is a common condition in COVID-19 hospitalized, which is characterized by increased troponin levels (59). Another definition of cardiac injury is reported as abnormality in cardiac biomarkers, electrocardiography, or echocardiography relative to the patient's previous condition. In a cohort study of 416 patients, 19.7% of hospitalized patients had a cardiac injury (34).

Cardiomyopathy was defined as evidence of new left ventricular systolic dysfunction on trans-thoracic echocardiography with one of the following criteria: 1. Clinical signs of cardiogenic shock, 2. Increase in creatine kinase or troponin level, and 3. Reduction in oxygen saturation of the central vein below 70% (43).

Our results showed that ARDS was present in 31.45% of patients following COVID-19 and cardiomyopathy. The cardiogenic shock occurred in 8% of patients. Reported data from Germany and the United States (56, 60) showed that cardiogenic shock is a significant complication of COVID-19. According to the evaluated studies in our systematic review, ~8% of patients developed heart failure/cardiogenic shock as a manifestation of COVID-19.

We showed that common symptoms of COVID-19 in patients with cardiac injury include fever, cough, headache, and fatigue. These findings are broadly consistent with other studies examining clinical signs in patients with COVID-19 (13, 14).

Our review of published studies showed the most common abnormal laboratory findings in patients with cardiomyopathy were increased IL-6 level, elevated ferritin, and High D-dimer. Some studies were reported that Serum concentrations of IL-6 were higher in severe cases of COVID-19 compared with moderate cases. Moreover, in deceased patients, levels of this cytokine were substantially higher than in recovered ones. So, continuous measurement of IL-6 level for early prediction of severity of infection has been suggested (61, 62).

The elevated level of fibrin degradation products, especially D-dimers (>2590/ng·mL^−1^), was shown to be an indicator of pulmonary embolism in hospitalized COVID-19 patients. It also contributed to poor prognosis and high mortality in patients with a more severe form of COVID-19 (63).

Our systematic review showed that 21.7% of patients presented with high troponin levels that were investigated in 14 studies. Gue et al. indicated the importance of monitoring troponin levels to predict the likelihood of cardiovascular events. Patients with high troponin levels had higher levels of other cardiac biomarkers and more fatal arrhythmias (34).

The results of our analysis revealed that hypertension, obesity, and hyperlipidemia were the most common comorbidities among patients with COVID-19. The association between hypertension and inflammation is well-known; inflammatory responses increase the disease's severity and complications in patients (64, 65). In a systematic review study, hypertension was the most common underlying condition in CMPs following COVID-19, reported in 33% of patients (66). Moreover, the presence of hyperinflammatory conditions in the airways interferes with the virus's clearance (67). It is inferred that the potential synergistic effect of inflammation due to hypertension and COVID-19 can aggravate this effect on the heart and result in CMPs.

Studies have shown that obesity is a risk factor for developing ARDS in COVID-19 (68). Moreover, hyperlipidemia has been more prevalent among hospitalized and more severe cases of COVID-19 compared to non-hospitalized ones (69, 70).

One of the common diagnostic modalities for COVID-19 is CT-scan. Bilateral and peripheral predominant ground-glass opacity, multifocal patchy consolidation, and interstitial changes with the peripheral distribution are among these features (71).

According to the included articles in our study, 76.47% of evaluated patients demonstrated Ground-glass opacities in their chest CT scan examination.

Different pharmacological and non-pharmacological treatments have been studied and applied for COVID-19. The included studies showed that nasal oxygen and intubation were among the most common non-pharmacological treatments for patients. Hydroxychloroquine, azithromycin, antiviral drugs, and β-Blockers were the most common pharmacological treatments. Due to the wide range of disease symptoms and complications, further studies related to each organ involvement are required to manage the disease better and prevent the complications.

In the end, it is necessary to point out the limitations of the present study. Since only case reports and case series studies have been selected for this review, this increases the potential risk of bias. Another issue is the small number of patients enrolled in the study. Due to the scarcity of randomized controlled trial (RCT)/quasi-randomized studies, we could not include them in the present study. We have not adopted the publications as abstracts or letters as data presented in this format is not high quality. Further investigations are required to include a broader range of studies, including clinical trials in patients with COVID-19 and CMPs.

In conclusion, cardiac injury and CMPs, including exacerbation of an underlying CMPs or the emergence of new CMPs, are common in COVID-19 patients. Moreover, they are associated with higher mortality and morbidity in these patients. Common fatal conditions in patients with COVID-19 CMPs include multiorgan damage, ARDS, and cardiogenic shock. Therefore, diagnostic measures of COVID-19 should consist of underlying cardiovascular comorbidities. History, signs, and symptoms of cardiac injury should be considered in evaluating these patients early in the course of this novel disease, and prompt therapeutic measures for the prevention of exacerbating cardiac condition should be sought.

## Data Availability Statement

The original contributions presented in the study are included in the article/supplementary material, further inquiries can be directed to the corresponding author/s.

## Author Contributions

FO, MN, and BH: designed the study. FO, SK, AT, SR, AA, SH, MG, and FK: performed the search, study selection, and data synthesis. BH, FO, and MN: wrote the first draft of the manuscript. MN, BH, and MM: revised the article. All authors contributed to the paper and approved the submitted version.

## Conflict of Interest

The authors declare that the research was conducted in the absence of any commercial or financial relationships that could be construed as a potential conflict of interest.
